# Resuspendable Powders of Lyophilized Chalcogen Particles with Activity against Microorganisms

**DOI:** 10.3390/antiox7020023

**Published:** 2018-01-27

**Authors:** Sharoon Griffin, Muhammad Sarfraz, Steffen F. Hartmann, Shashank Reddy Pinnapireddy, Muhammad Jawad Nasim, Udo Bakowsky, Cornelia M. Keck, Claus Jacob

**Affiliations:** 1 Division of Bioorganic Chemistry, School of Pharmacy, Saarland University, D-66123 Saarbruecken, Germany; sharoon.griffin@uni-saarland.de (S.G.); s8musarf@stud.uni-saarland.de (M.S.); jawad.nasim@uni-saarland.de (M.J.N.); 2 Department of Pharmaceutics and Biopharmaceutics, University of Marburg, 35037 Marburg, Germany; steffenfhartmann@googlemail.com (S.F.H.); shashank.pinnapireddy@pharmazie.uni-marburg.de (S.R.P.); ubakowsky@aol.com (U.B.)

**Keywords:** antimicrobial activity, chalcogen nanoparticles, mannitol, resuspended lyophilized nanosuspensions (NaLyRe), sulfur, selenium, tellurium

## Abstract

Many organic sulfur, selenium and tellurium compounds show considerable activity against microorganisms, including bacteria and fungi. This pronounced activity is often due to the specific, oxidizing redox behavior of the chalcogen-chalcogen bond present in such molecules. Interestingly, similar chalcogen-chalcogen motifs are also found in the elemental forms of these elements, and while those materials are insoluble in aqueous media, it has recently been possible to unlock their biological activities using naturally produced or homogenized suspensions of respective chalcogen nanoparticles. Those suspensions can be employed readily and often effectively against common pathogenic microorganisms, still their practical uses are limited as such suspensions are difficult to transport, store and apply. Using mannitol as stabilizer, it is now possible to lyophilize such suspensions to produce solid forms of the nanoparticles, which upon resuspension in water essentially retain their initial size and exhibit considerable biological activity. The sequence of Nanosizing, Lyophilization and Resuspension (NaLyRe) eventually provides access to a range of lyophilized materials which may be considered as easy-to-handle, ready-to-use and at the same time as bioavailable, active forms of otherwise insoluble or sparingly substances. In the case of elemental sulfur, selenium and tellurium, this approach promises wider practical applications, for instance in the medical or agricultural arena.

## 1. Introduction

Organic chalcogen compounds have been investigated for several decades due to their often pronounced biological activities against parasites, microorganisms and cancer cells. Indeed, the field of natural Organic Sulfur Compounds (OSCs) has attracted tremendous attention, as Reactive Sulfur Species (RSS) such as allicin and polysulfanes from garlic or allylisocyanate (mustard oil) from various *Brassica* plants exhibit interesting chemopreventive and possibly even therapeutic properties [1]. Organoselenium compounds, in particular ebselen and its derivatives, pose a similar attraction in the Life Sciences, especially in the context of antioxidants [2,3]. In contrast, organotellurium compounds have long remained marginalized in Biology, yet some of them, such as RT-01 and AS101, have also recently emerged from the chemical closet with some prominence [2,4,5,6]. Not surprisingly, the field of biologically active organochalcogens has stimulated considerable efforts in chemical synthesis and has produced volumes of more or less exotic molecules [7,8].

One of the predominant modes of action of many of these compounds is due to their ability to oxidize—rather selectively—the thiol groups of cysteine residues in pivotal cellular signalling proteins and enzymes, hence triggering processes which eventually may culminate in an antioxidant response, or the initiation of apoptosis or other forms of cell death. Intriguingly, the chalcogen-chalcogen bond often at the centre of such redox action is also found in the elemental forms of sulfur (S_8_, common yellow form), selenium (Se_8_, red allotrope) and tellurium (Te_8_, dark grey), implying that such elemental modifications, from the perspective of reactivity, may themselves also be biologically active [9]. Indeed, recent studies on inorganic polysulfides, such as the tetrasulfide S_4_^2−^, support this notion of simple, purely inorganic, “carbon free” yet highly active variants of chalcogens [10,11,12]. Unfortunately, neither of the various elemental forms of any of the three chalcogens in question is soluble in water. Hence traditionally it has mostly been futile to apply elemental sulfur, selenium or tellurium in a biological context, with some notable exceptions, such as colloidal sulfur and its uses as fungicide in vineyards [13,14].

Indeed, the apparent lack of solubility and hence bioavailability can be overcome with suspensions of small particles with diameters in the low or sub-micrometer range. We have recently shown that such particles can be obtained rather easily by physical or chemical methods and, in the case of selenium, even with the assistance of certain bacteria which readily generate natural, protein-coated selenium nanoparticles in adequate quality and yield. Most of these particles show some activity against microorganisms [15]. Nanosized tellurium, in particular, appears to be rather active against organisms such as *Escherichia coli*, *Candida albicans*, *Saccaromyces cerevisiae* and the model nematode *Steinernema feltiae* [16]. While the nanosuspensions employed as part of such studies are rather promising as a first proof-of-concept for production and activity, they also have considerable drawbacks once used in practice. For instance, such liquid suspensions are difficult to maintain and to store for prolonged periods of time, they need to be kept under sterile conditions, are not particularly amenable to transport, and an adjustment of reliable concentrations is generally difficult. We have therefore undertaken a series of studies to produce easier-to-handle solid materials based on elemental chalcogens. Here we report our first results with the underlying NaLyre sequence of processing, which involves nanosizing, then lyophilizing and subsequently resuspending mannitol-stabilized chalcogen nanoparticles, and biological activities associated with them.

## 2. Materials and Methods

### 2.1. Production of Chalcogen Nanoparticles

Chalcogens were purchased from the following companies: Sulfur from Carl Roth GmbH + Co. KG (Karlsruhe, Germany), selenium from ThermoFisher Acros Organics (Geel, Belgium) and tellurium from Sigma-Aldrich Chemie GmbH (Taufkirchen, Germany). For every chalcogen, suspensions in distilled water were produced containing 1% of the respective chalcogen as well as 1% of Plantacare**^®^** 2000 UP (BASF, Ludwigshafen, Germany) as particle stabilizer. The coarse suspension was produced by dispersing the chalcogen into the surfactant solution under magnetic stirring. These suspensions were stored in the refrigerator at 4 °C until further use. Prior to High Pressure Homogenization (HPH) the coarse suspensions were stirred using a Polytron**^®^** PT2100 high-speed stirrer (Kinematica GmbH, Luzern, Switzerland) attached to a Polytron**^®^** PT-DA-2112/EC aggregate. Each sample was stirred ten times at 11,000 rpm for 1 min each, a technique known as High Speed Stirring (HSS). Afterwards HPH was performed using an APV Gaulin Lab 40 high pressure homogenizer (APV Deutschland GmbH, Unna, Germany). Initial pre-milling involved 3 cycles each at 250 bar, 500 bar, 750 bar and 1000 bar, respectively. Subsequent main milling consisted of 10 cycles at 1500 bar. Between each cycle the sample was cooled to below 5 °C to guarantee stability and to ensure that the temperature during milling remained below 30 °C. The physical stability of those particles was monitored over time using a combination of analytical techniques routinely employed for size characterization (see Section 2.3). Thereafter, the nanosuspensions were stored at a refrigerator at 4 °C for 30 days and inspected at day 1 (1 day after production) and finally at day 30 after production.

### 2.2. Lyophilization of Chalcogen Nanoparticles

As part of the lyophilization studies, the appropriate concentration of mannitol (ThermoFisher Acros Organics) was determined first using the selenium nanosuspension. Mannitol, in particular, was selected as it is a popular choice of “cryoprotectant” for freeze-drying of nanoparticles [17,18,19]. Different concentrations of mannitol (0.01 g, 0.02 g, 0.05 g and 1 g) were added to 5 mL of selenium nanosuspension to yield suspensions with 1%, 2%, 5% and 20% *w*/*v* of mannitol. Together with a 5 mL sample without any mannitol (0%), these five samples were lyophilized using an Alpha 1-4 LSC lyophilizer (Martin Christ Gefriertrocknungsanlagen GmbH, Osterode am Harz, Germany). The freeze-drying steps were performed in the following manner: Freezing (−80 °C, overnight), main drying (−50 °C, 0.120 mbar for 48 h) and final drying (25 °C, above 1 mbar pressure for 24 h). The conditions for lyophilization were optimized for mannitol according to the literature with appropriate modifications [17,18,20]. Eventually, the 20% suspension was deemed effective for achieving the respective sizes after freeze-drying. Samples of sulfur and tellurium nanosuspensions were then lyophilized under the same conditions.

### 2.3. Size Characterization of Chalcogen Nanoparticles

The samples prior to lyophilization (see above) as well as samples of 1.05 g of lyophilized material re-suspended in 5 mL of distilled water were analyzed with regard to their size, size distribution and particle morphology, using Photon Correlation Spectroscopy (PCS, also known as Dynamic Light Scattering), Laser Diffraction (LD, also known as Static Light Scattering) and Light Microscopy (LM). PCS measurements were performed using a Zetasizer Nano ZS (Malvern Instruments, Worcestershire, UK). Prior to the measurements samples were diluted by a factor 1:200. All measurements were performed at 21 °C. LD was performed on a Mastersizer 3000 (Malvern Instruments, UK) attached with a HydroMV dispersion unit. Mie theory was applied by using the optical parameter (real/imaginary refractive index) 1.570/0.010. For the morphological analysis an Olympus BX53 light microscope (Olympus Cooperation, Tokyo, Japan) with an Olympus SC50 CMOS color camera was employed (Olympus soft imaging solutions GmbH, Muenster, Germany).

### 2.4. Zeta Potential (ZP) Measurements of Chalcogen Nanoparticles

The charge of the chalcogen nanoparticles was assessed via ZP measurements using a Malvern Zetasizer Nano ZS (Malvern Instruments, UK) at 21 °C and a field strength of 20 V/cm. ZP measurements via the laser Doppler anemometry yield the electrophoretic mobility, which was converted to the ZP by using the Helmholtz–Smoluchowski equation. ZP measurements were performed in conductivity-adjusted Milli-Q water (50 S/cm, using 0.9% NaCl solution) and in the original surfactant solution [21]. The measurements in the original solution are important for predicting long term stability, while the measurements in conductivity-adjusted water are indicative of the potential at the Stern layer. Differences in these measurements can be used, among others, to predict the ability of the surfactant to bind to the surface of the particles [22,23].

### 2.5. Scanning Electron Microscopy (SEM) of Chalcogen Nanoparticles

Scanning electron microscopy (SEM) was performed using a Hitachi S-510 Scanning Electron Microscope (Hitachi-High Technologies Europe GmbH, Krefeld, Germany). The nanoparticle suspensions were pipetted onto SEM pin stubs fixed with conductive carbon tabs and air dried. The samples were then sputtered with gold at 13.3 Pa argon and 30 mA using an Edwards S150 sputter coater (Edwards Vacuum, Crawley, UK) and examined using SEM at an accelerating voltage of 5 kV and 30 µA emission current under 4.6 × 10^−4^ Pa vacuum [24]. The micrographs were recorded digitally using the DISS 5 digital image acquisition system (Point Electronic GmbH, Halle/Saale, Germany).

### 2.6. Nematicidal Activity of Chalcogen Nanoparticles

The model nematode *S. feltiae* was purchased from Sautter und Stepper GmbH (Ammerbuch, Germany) in the form of powder and stored at 4 °C in the dark. Fresh samples were used prior to each experiment. A homogeneous mixture was prepared by dissolving 200 mg of nematode powder in 50 mL distilled water. Later on, the nematode suspension was placed for 30 min at room temperature with occasional shaking and in moderate light. Viability was examined under a light microscope at four-fold magnification (TR 200, VWR International, Leuven, Belgium). Viability of nematodes above 80% in each sample was considered as prerequisite for each experiment.

10 µL of nematode suspension were added to each well of a 96-well plate. Suspensions of the nanosized samples (sulfur, selenium and tellurium) were then added into the wells to achieve final concentrations of 25, 50, 100 and 200 µM. Afterwards, the final volume in each well was adjusted to 100 µL by adding Phosphate Buffered Saline (PBS pH = 7.4). PBS and ethanol (10 µL per well) were used as negative and positive control, respectively. Each experiment was performed independently on three different occasions and in triplicate (*n* = 9). Living and dead nematodes were counted under the microscope prior to treatment, and the viability fraction (V_0_) was calculated (usually > 0.9). After 24 h, the V_24_ fraction was calculated by once more counting the living and dead nematodes. 50 µL of lukewarm water (50 °C) was added to each well to stimulate the nematodes prior to counting. After 24 h, the viability was calculated and expressed as a percentage of initial viability V_0_ according to Equation (1):

Viability (%) = [V_24_/V_0_] × 100 (1)

Results are represented as mean ± SD and GraphPad Prism (Version 5.03, GraphPad Software, La Jolla, CA, USA) has been used to calculate the statistical significances by one-way ANOVA, with *p* < 0.05 considered to be statistically significant.

### 2.7. Antimicrobial Activity of Chalcogen Nanoparticles

The activity of nanosized suspensions of sulfur, selenium and tellurium against *E. coli*, *Staphylococcus carnosus*, *C. albicans* and *S. cerevisiae* was investigated in routine microbial growth assays based on optical density and recorded in the form of growth curves [15]. Fresh cultures of *S. carnosus*, *E. coli*, *C. albicans* and *S. cerevisiae* were prepared on bacterial basic media, Luria-Bertani broth (LB) Sabouraud Dextrose Agar (SDA) and Yeast Peptone Dextrose (YPD) agar media, respectively. After 18–24 h of incubation, the microbial colonies from these plates were used to inoculate liquid cultures. Liquid cultures were subsequently incubated at 37 °C until reaching an optical density of 0.8–1.0. These microbial cultures were then exposed to the nanoparticles as described below. Bacterial or yeast culture with growth medium was employed as negative control while the positive control was composed of a mixture of penicillin, streptomycin and amphotericin B (4 U, 0.4 µg/mL and 10 µg/mL, respectively). An additional solvent control—which was required here because of the presence of stabilizers—consisted of a mixture of mannitol (20%) and Plantacare^®^ (1%) whose concentrations were adjusted according to the one present in the sample. Nanosized suspensions were evaluated at various dilutions (formal chalcogen concentrations of 750, 1000, 1500 and 2000 µM) and the plates were incubated at 37 °C for 24 h. It should be mentioned upfront that these are formal concentrations as the particles do not dissolve (see Results). Microbial as well as fungal growth both were monitored by recording the optical density of the samples (A_540_) at 0, 4, and 24 h (0 h refers to the first measurement immediately after incubation) on a Cary50 Bio UV/VIS spectrophotometer (Varian Australia Pty Ltd., Mulgrave, Australia). These absorbance values were converted to percentage values compared to the negative control whose value was used as a reference at each time interval and set at 100%, respectively. In essence, growth (%) represents the percentage of UV-Vis spectrophotometric absorbance values compared to the negative control.

As some of the particle suspensions themselves also absorb or scatter light, nanosuspensions without microorganisms were used as additional controls to adjust the readings accordingly. All experiments were carried out in triplicate, at three different occasions (*n* = 9). Results are represented as mean ± SD and statistical significances have been calculated by two-way ANOVA using GraphPad Prism (Version 5.03, GraphPad Software, La Jolla, CA, USA) with *p* < 0.05 considered to be statistically significant.

## 3. Results

Overall, the results obtained as part of this study support the idea that suspensions of stabilized chalcogen-nanoparticles can be lyophilized to yield ready-to-use powders which can be resuspended on demand without notable loss of particle quality (i.e., no significant aggregation) and with a range of interesting biological activities against common pathogens such as *E. coli*, *S. carnosus* and *C. albicans*. These results will now be presented and discussed in more detail.

### 3.1. Nanosizing, Lyophilization and Resuspension (NaLyRe) of Chalcogen Particles

**Figure 1:**
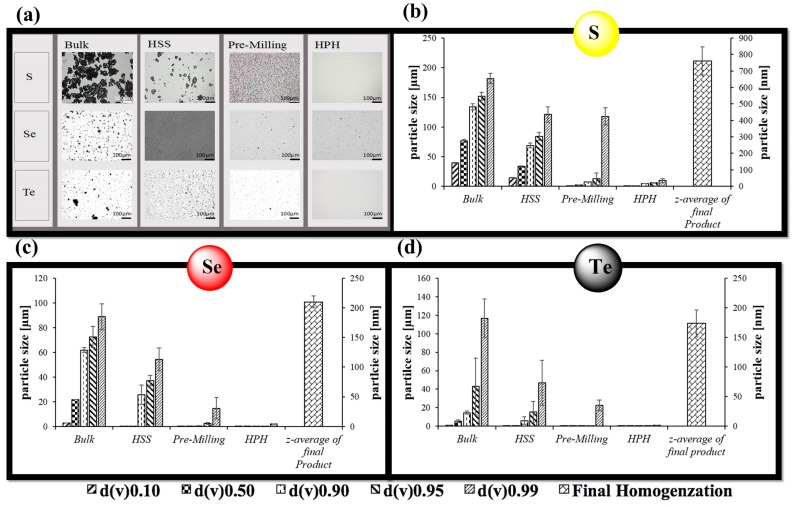
Characterization of nanosized chalcogen particle suspensions. (**a**) 200× magnification of nanoparticles at different generation steps; LD and PCS measurements for (**b**) sulfur; (**c**) selenium and (**d**) tellurium. It should be noted that light microscopy has been used here primarily to monitor the nanosizing process and per se is not an adequate technique to characterize particles in the nanosize range.

In good agreement with our previous findings, a combination of HSS, pre-milling and HPH resulted in spherical particles of sulfur, selenium and tellurium with diameters in the range of 760 nm for sulfur, 210 nm for selenium and 175 nm for tellurium (Figure 1) [15,25]. Figure 1 confirms that the combination of so-called pre-milling and subsequent cycles of HPH is able to reduce the size of the particles considerably. A lower limit is reached in all cases, implying that the method at hand is able to produce good quality particles with sub-micrometer diameters, yet cannot go much below the 100 nm range.

**Figure 2:**
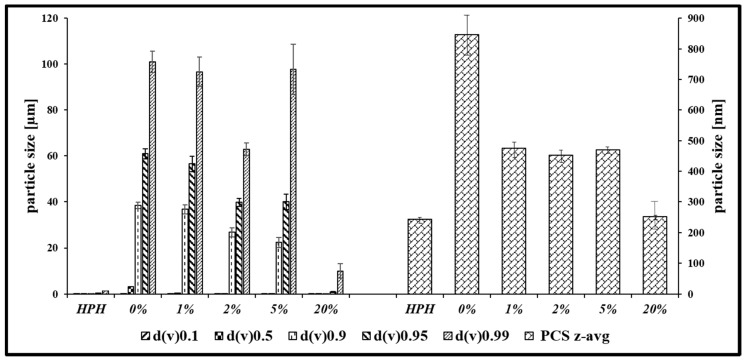
Determination of the effective concentration of mannitol for stabilizing selenium nanoparticles during lyophilization. LD and PCS measurements of selenium nanoparticles at 0%, 1%, 2%, 5% and 20% *w*/*v* of mannitol are shown. The HPH sample did not undergo freeze-drying and its quality, which serves as a benchmark, is achieved in the freeze-dried samples in the presence of 20% *w*/*v* mannitol.

The nanosized suspensions shown in Figure 1 are of adequate quality and can be employed for biological studies soon after production. Nonetheless, a prolonged storage may result in aggregation, decomposition or other detrimental events. While the manufacture of such suspensions under high pressure ensures that they are initially sterile, contaminations are common during handling and result in fouling of such samples. Lyophilization in the presence of the well-known cryoprotectant mannitol was therefore seen as a suitable alternative to produce low weight, durable, readily storable and transportable samples. Figure 2 illustrates the ability of mannitol to help prevent possible agglomeration upon freeze-drying, which is clearly concentration depended. In the presence of about 20% *w*/*v* of mannitol, the size of particles remains comparably unaffected by a sequence of freeze-drying and subsequent resuspension in distilled water, with an average diameter of 245 nm before and 250 nm afterwards.

Once the most appropriate concentration of mannitol as “cryoprotective” was determined, it was applied to the various sulfur, selenium and tellurium suspensions. After freeze-drying, these powder-like samples were stored at 4 °C and then, on demand, resuspended in 5 mL of distilled water by gentle manual shaking for 1 min. The freeze-dried samples could be stored as powders for 30 days in our experiments without notable physical changes.

**Figure 3:**
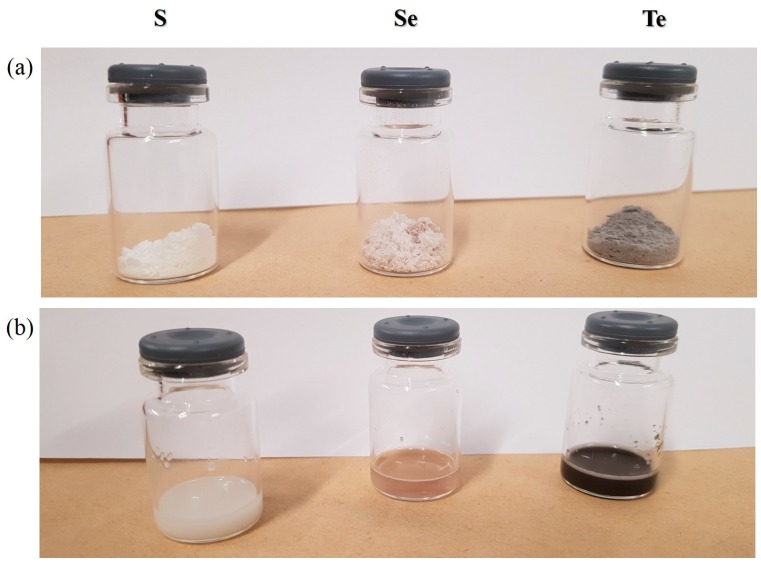
Photographic view of nanosized chalcogens. (**a**) lyophilized powders; (**b**) resuspensions after addition of water and simple manual shaking for 1 min.

Figure 3 shows photographic images of the lyophilized samples before and after resuspension in distilled water. In essence, the lyophilized samples shown in Figure 3a are powder-like and show the corresponding colors of the respective nanosized materials somewhat eclipsed by the white color of mannitol, i.e., sulfur (pale yellow), selenium (light pinkish red) and tellurium (grey) (Figure 3a). In some aspects, these materials represent “resuspendable” and biologically available forms of the three elements, which apart from the stabilizers Plantacare^®^ (for particle stability) and mannitol (for freeze-drying) consist *exclusively* of the chalcogen elements. Unlike the elements they are based on, however, these materials can be resuspended readily and easily as shown in Figure 3b.

**Figure 4:**
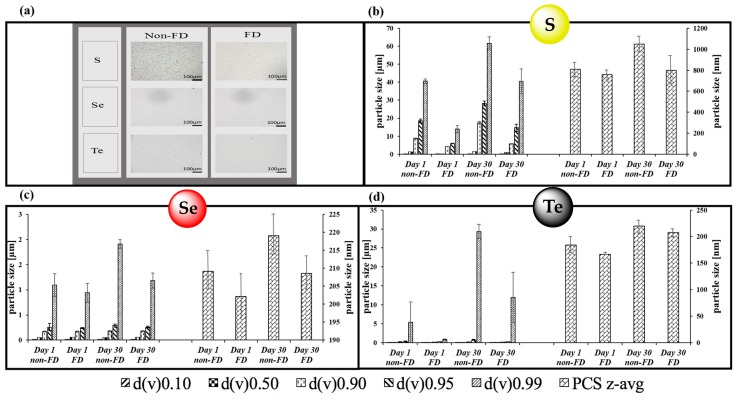
Analysis of the various chalcogen samples. (**a**) Microscopic view of samples after 30 days of storage, with a comparison of samples stored as suspensions at 4 °C in the dark (non-FD or “non-freeze-dried”) and resuspended lyophilized samples (FD or “freeze-dried”); LD and PCS measurements of resuspended lyophilized samples of (**b**) sulfur, (**c**) selenium and (**d**) tellurium samples after 1 and 30 days of storage. The values obtained compare well with the results for the native samples provided in Figure 1.

The native samples before lyophilisation and resuspended samples were then inspected more closely for their stability at storage conditions (at 4 °C and in a dark environment). Here, the average particle size as well as size distribution was monitored employing LD and PCS and with a firm focus on possible aggregation. Figure 4 confirms that the samples stored for 30 days at 4 °C as well as the lyophilized and subsequently resuspended samples both in their liquid forms retained the main physical characteristics of the original, freshly nanosized and pre-lyophilized samples at Day 1, such as average size and size distribution. Indeed, it appears that all native chalcogen suspensions (Figure 4a), re-suspensions (Figure 4a–d) as well as the freeze-dried powders (Figure 3a) were reasonably stable for well over one month at 4 °C, with some possible exceptions, which showed some larger aggregates, as noted with LD. Yet here as well, those larger particles seemed to be the exception as the average diameter did not increase significantly and overall, the suspensions also proved quite stable and without large agglomerates detectable under the light microscope.

Furthermore, to estimate the long-term stability of the nanosuspensions, measurements of their respective ZPs were performed. The ZP is an indicator of electrokinetic potential and hence can be used to predict the stability of colloidal dispersions. As mentioned already, ZPs were measured in the original suspension medium as well as in conductivity-adjusted Milli-Q water. The measurements in conductivity-adjusted Milli-Q water and in original dispersion medium were important to provide a larger picture of the charges and their effects on stability. The Stern potential, as estimated through measurements in conductivity-adjusted water, corresponds to the potential at the Stern layer and is indicative of possible electrodynamic attractions or repulsions.

The values for the initial nanosuspensions as well as the resuspended lyophilized samples are provided in Table 1.

The ZPs measured in this medium range between −31 mV and −47 mV in the samples before freeze-drying for tellurium and sulfur, respectively, and between −36 mV and −54 mV for tellurium and sulfur, respectively, in the samples after freeze-drying, with selenium at −40 mV before and −41 mV after freeze-drying. These values correspond to the respective Stern potentials and indicate well-charged particles, whose negative charges in turn prevent aggregation and therefore promote stability. The ZP measurements in original dispersion medium (surfactant and water), provide another perspective on stability. In this instance, the ZPs relate directly to the stabilization efficacy of the particles in the original suspension [22,23]. The chalogen nanosuspensions in the original medium show ZPs between −27 mV to −30 mV for tellurium and sulfur, respectively, before freeze-drying, and between −30 mV to −36 mV for tellurium and sulfur, respectively after freeze-drying, with selenium at −24 mV before and −26 mV after freeze-drying. Overall, ZP values below −30 mV are normally considered adequate for long-term physical stability. Here, one must also take into account that ZPs are not only dependent on the characteristics of the substance but also on the surfactants used. Plantacare^®^ used in the present study is a non-ionic sugar-based surfactant [26]. Future studies should include a variety of surfactants in order to learn more about the surface charges and their impact on the stability of chalcogens. In a nutshell, the particles are charged enough to repel each other and hence do not aggregate and the sequence of lyophilizing and resuspension does not change this aspect dramatically. If at all, these processes may result in a small increase in negative charge by a few millivolts (see Table 1).

**Figure 5:**
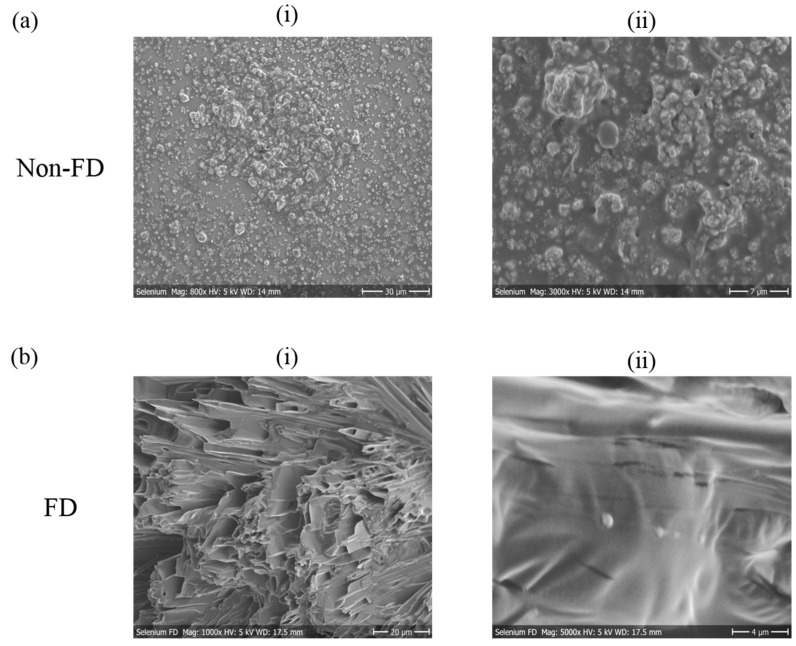
SEM micrographs of selenium particles. (**a**) micrographs of non-FD samples at (i) 800 and (ii) 3000× magnification; (**b**) micrographs of FD samples at (i) 1000× and (ii) 5000× magnification.

Another technique for visualizing the stability of nanoparticles is microscopy of suspensions over time. Since the size of particles was out of the range and reach of light microscopy, SEM imagery was employed [24]. As described earlier (Section 2.5), SEM requires drying of suspensions, which results in extensive agglomeration for the sample before freeze-drying (Figure 5a). This complication was avoided with the freeze-dried samples as these samples were lyophilized in the presence of a cryoprotectant (Figure 5b). Nonetheless, the presence of this cryoprotectant, which is also “dried”, complicates the images obtained. The micrographs therefore show the mannitol matrix (Figure 5b (i)) and the selenium particles embedded therein (Figure 5b (ii)). Please note that the samples are dried and hence the presence of slats, composed of mannitol, complicates the images and also results in apparent artefacts.

### 3.2. Antimicrobial Activity of Resuspended Samples

The lyophilized samples shown in Figure 3a were resuspended on demand to yield suspensions of defined concentrations. These suspensions were then investigated for their potential antimicrobial and nematicidal activity in assays based on Gram-negative (*E. coli*) and Gram-positive bacteria (*S. carnosus*), yeasts (*S. cerevisiae* and *C. albicans*) and eventually the model nematode *S. feltiae* [15,25]. It must be emphasized from the outset that the concentrations given (in molar units) are for the entire amount of the respective chalcogen in suspension. As the nanoparticles obviously do not dissolve, their chemical action is restricted to the chalcogen atoms exposed to the particle surface, and therefore also critically depends on the shape and size of the particles in question. Hence, as a rough estimate, only between 0.1% and 1% of the total chalcogen atoms are likely to be exposed and able to participate in biological activity, assuming the particles are spherical, smooth and around 100 nm in diameter. This implies, however, that formal “concentrations” shown in the following figures and discussed in the text cannot be compared directly with completely soluble materials as they obviously have to be considerably higher in order to exert the same chemical reactivity and biological activity (see Discussion).

**Figure 6:**
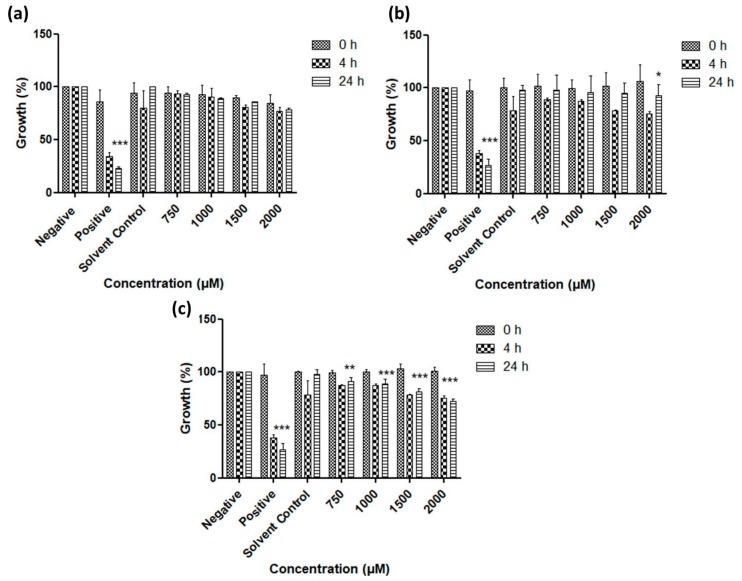
Impact of resuspended nanosized particles of (**a**) sulfur, (**b**) selenium and (**c**) tellurium on the growth of *S. cerevisiae*. Values represent mean ± S.D. * *p* < 0.05, ** *p* < 0.01 and *** *p* < 0.001. See text for further experimental details.

**Figure 7:**
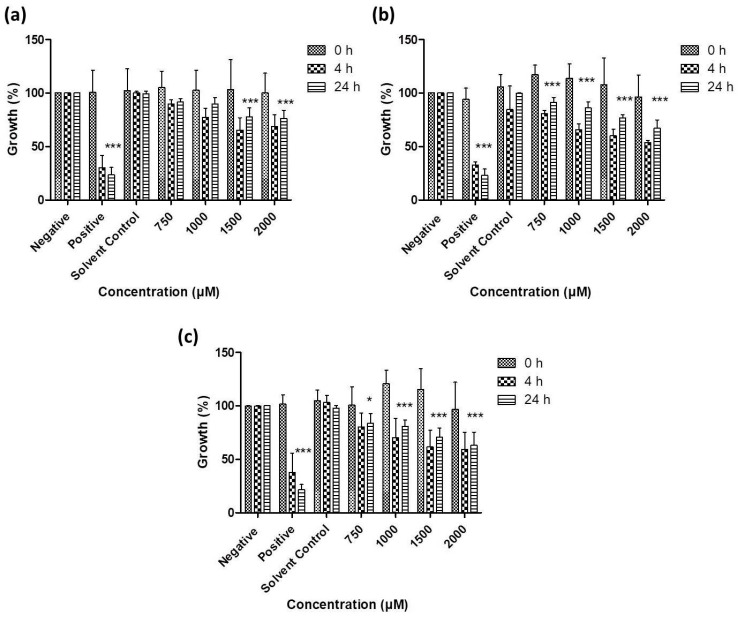
Impact of resuspended nanosized particles of (**a**) sulfur (**b**) selenium and (**c**) tellurium, on the growth of *C. albicans*. Values represent mean ± S.D. * p < 0.05 and *** p < 0.001. See text for further details.

**Figure 8:**
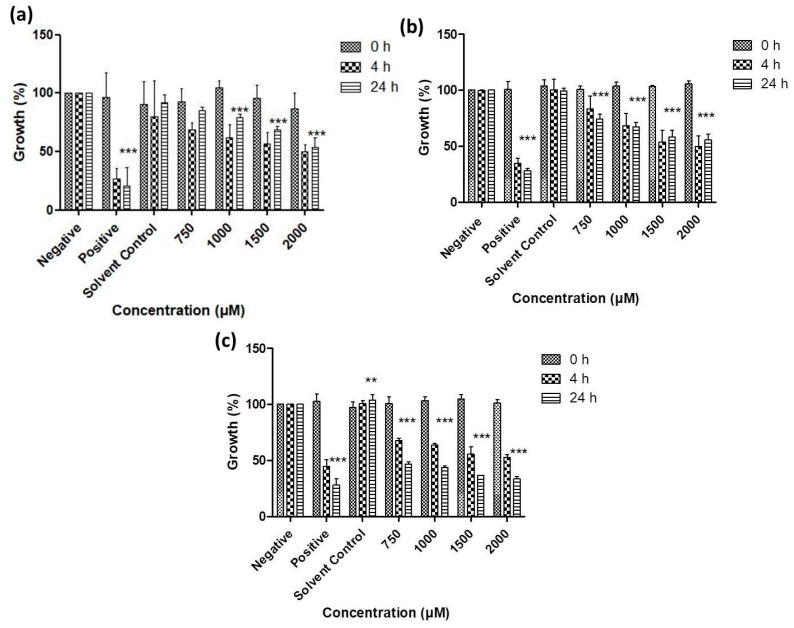
Impact of resuspended nanosized particles of (**a**) sulfur, (**b**) selenium and (**c**) tellurium on the growth of *E. coli*. Values represent mean ± S.D. ** p < 0.01 and *** p < 0.001. See text for further details.

**Figure 9:**
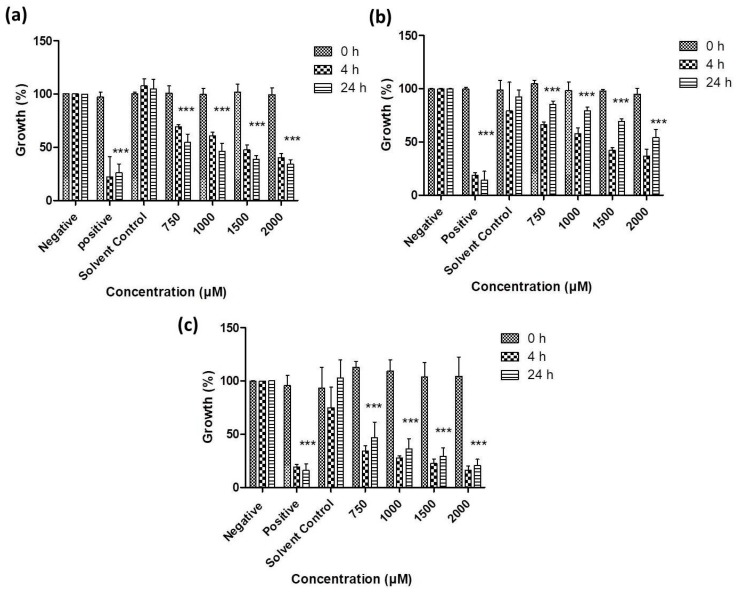
Impact of lyophilized nanosized particles of (**a**) sulfur, (**b**) selenium and (**c**) tellurium on the growth of *S. carnosus*. Values represent mean ± S.D. *** p < 0.001. See text for further details.

As Figure 6, Figure 7, Figure 8 and Figure 9 illustrate, overall there is a notable toxicity of the (re-)suspended chalcogen particles against most microorganisms under investigation (*S. cerevisiae, C. albicans, E. coli*, *S. carnosus* and *C. albicans*). Generally, this toxicity is concentration-dependent and can be noted after 4 h. After 24 h, growth remains reduced in most cases when compared to the control, and in some cases there may even be a slight “recovery” of growth, probably due to the surviving cells which continue or resume their growth.

Baker’s yeast exemplifies several aspects of this activity (Figure 6). The growth of *S. cerevisiae* is affected particularly by the tellurium particles, especially at higher concentrations, with some activity also noted for the selenium particles. Still, *S. cerevisiae* is rather resilient towards exposure to these particles, especially in the case of sulfur and selenium.

In contrast, the growth of the pathogenic yeast *C. albicans* is affected significantly by all three types of chalcogen particles. In the case of sulfur particles, a modest reduction in growth by 20% to 30% can be noted after 4 h (Figure 7). The selenium particles are even more active and able to reduce viability by almost 55% at formal concentrations of around 1 mM of total selenium in suspension. The activity of tellurium particles is comparable, possibly slightly lower when compared to selenium.

Gram-negative bacteria currently pose a particular challenge in the development of effective antibiotics, and Figure 8 indicates that resuspended samples of all three chalcogens are active against *E. coli* at formal chalcogen concentrations in the higher micromolar range. Not surprisingly, the rather toxic tellurium is more active when compared to selenium and sulfur, with a reduction in growth to less than 50% (vs. control) after 24 h incubation at 750 μM, while around 75% of growth is retained when the same concentration of selenium is applied. The sulfur nanoparticles are less active and achieve a similar reduction in growth only at higher concentrations of 1000 to 2000 μM.

A similar trend is also observed in the case of the Gram-positive bacterium *S. carnosus* (Figure 9). At a formal tellurium concentration of 750 μM, the tellurium particles reduce growth to less than 40% after just 4 h, while selenium and sulfur both are somewhat less active, with a remaining growth of around 70% in both cases. Indeed, higher formal concentrations of tellurium particles impact dramatically on the growth and survival of *S. carnosus*, with just 20% to 30% growth remaining at formal tellurium concentrations of and above 1000 μM. Once more, it is likely that the active concentration of truly available, surface-exposed tellurium in those samples is considerably lower, probably in the order of 1 to 10 μM at most.

### 3.3. Activity against S. Feltiae

**Figure 10:**
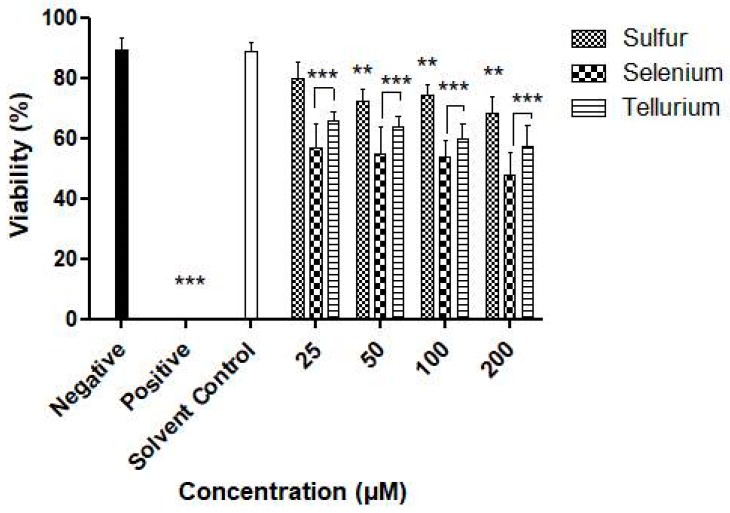
Impact of resuspended nanosized particles of sulfur, selenium and tellurium against *S. feltiae*. Values represent mean ± S.D. ** p < 0.01 and *** p < 0.001. See text for further details.

So far, the investigation has focused on the activity of the nanosuspensions against single cell organisms. In this, case, uptake, transport, metabolism and excretion differ considerably from multicellular organisms, which on the one side may be more protected against such particles yet, at the same time, may also become impaired by the particular particulate nature of such insoluble objects. The agricultural nematode *S. feltiae* has therefore been employed since it represents a readily available, easy to use, robust and fairly reliable model system to study toxicity against a small, yet multicellular organism [27,28,29]. The results for the different chalcogen particles are shown in Figure 10. In essence, they agree rather well with the previous findings in *C. albicans* and bacteria. It appears that the tellurium and selenium particles at concentrations from 25 μM upwards are more active when compared to the sulfur ones. After 4 h and at a concentration of 25 µM, a reduction in viability by 21%, 44% and 35% is observed for the sulfur, selenium and tellurium particles, respectively, which at 50 µM increases slightly to 28%, 46% and 37%, respectively. This toxicity, studied at lower concentrations due to a particular sensitivity of the nematodes against the chalcogens when compared to yeasts and bacteria, is indeed more pronounced as in the case of these microorganisms. Regardless of the chalcogen investigated, there seems to be some overall toxicity against the nematodes, which obviously needs to be investigated further and in considerably more detail, also with a sight on the impact of the stabilizers Plantacare^®^ and mannitol which have been employed throughout as additional solvent controls.

## 4. Discussion

Overall, the results obtained as part of this feasibility study demonstrate that a sequence of nanosizing and lyophilization leads to ready-to-use materials which can be resuspended easily by short manual shaking to nanosuspensions with improved bioavailability. In this study, such a NaLyRe sequence results in nanosuspensions of sulfur, selenium and tellurium, which are generally of a good physical quality and show impressive activity against a range of microorganisms, notably *C. albicans*, *E. coli* and *S. carnosus*. The results obtained along this NaLyRe avenue and their implications will now be discussed in more detail.

From the perspective of sample preparation and quality, formation of the various chalcogen nanosuspensions through mechanical techniques such as HPH was generally straightforward. Still there were also some notable differences (Figure 1). Sulfur was the least amenable of the three elements, its tendency to form cake-like materials and to sediment could be decreased by nanosizing, yet the average size of the sulfur particles could not be reduced much below 760 nm. Moving down the Periodic Table, the ability to nanosize seems to improve while applying the same experimental parameters. Under these conditions, selenium could be sized to an average particle diameter of 210 nm, while tellurium particles showed an average diameter of 170 nm. Similarly, the ability to lyophilize and to resuspend was most pronounced for the selenium and tellurium particles, while the resuspensions of the sulfur particles were stable, yet also rather “milky” (Figure 3).

**Figure 11:**
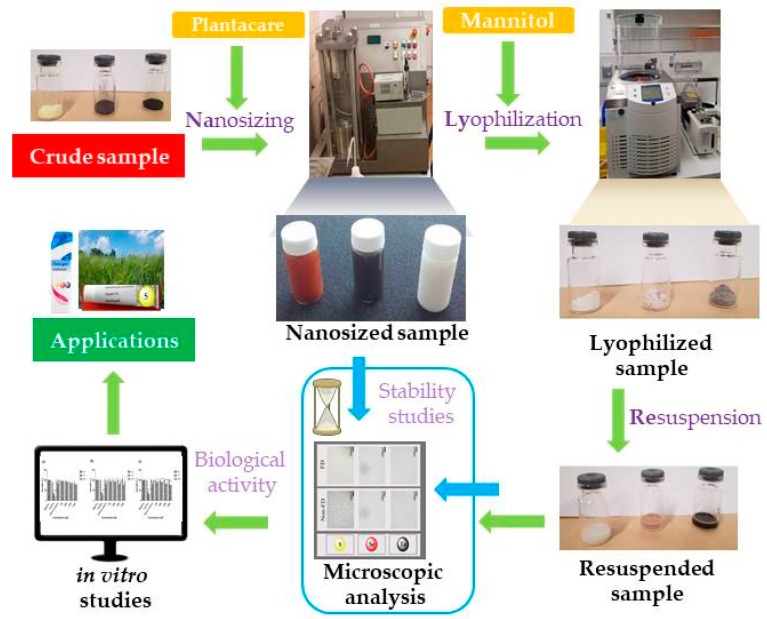
Schematic overview of the NaLyResequence to nanosize chalcogens and similar insoluble materials to nanosuspensions, to lyophilize these suspensions and store the resulting powders for possible practical applications. The biological activity associated with the resuspended samples promises possible applications in various areas, from Medicine to Agriculture.

From a more practical and applied perspective, these findings are rather intriguing as they represent a strategy to convert solid elemental sulfur, selenium and tellurium into powders of a similar elemental composition, yet with good suspension properties and hence applicability and activity in biological systems. Once lyophilized, the chalcogen nanoparticles form easy-to-handle fluffy cakes (Figure 3a) which require short manual resuspension times and maintain the size characteristics of the origin suspensions (Figure 3 and Figure 4). Indeed, it appears that most particles retain their initial size and shape during the lyophilization/resuspension process, and that aggregation is not an encumbering issue. The respective ZPs, employed here as indicators of the stability of colloidal dispersions, also remain mostly unaffected by the lyophilization and resuspension procedure, and may even increase slightly. Unlike earlier liquid preparations, these lyophilized powders are considerably lighter, easier to store and to transport and do not need to be used up swiftly as they are dry and there is no danger of fouling. There is also no issue with leaks or spills which are common when handling liquid samples. Eventually, it is now feasible to prepare and store larger quantities of chalcogen nanoparticles of good quality and to resuspended them if, when and where desired for a range of possible applications, for instance in the fields of Medicine, Agriculture, Cosmetics and conceivably even in Nutrition, as may be applicable for selenium. Figure 11 provides a brief schematic illustration of the NaLyRe sequence and the potential medium-term applications associated with it.

Still, there are some issues which need to be addressed as part of subsequent studies. One of them is the use of stabilizers such as Plantacare^®^ to prevent aggregation in solution and mannitol as “cryoprotectant”. These components of the formulation are critical in providing protection against the stresses involved during the freeze-drying process. Indeed, without such stabilizers the formulation may be damaged in two ways. Firstly, particles at higher concentrations tend to agglomerate and to fuse. Secondly, the formation of ice crystals exerts mechanical stresses which destabilize the system [30]. These effects in the absence of protective materials were, in fact, observed in this study as illustrated in Figure 2. Here, the choice and concentration of protectant, as well as the size of particles affected are of importance. At concentrations of mannitol ranging from 1% to 5%, LD measurements show little impact of this apparent “cryoprotectant” on the agglomeration of larger particles. At higher concentrations of mannitol (e.g., at 20%), the integrity of the smaller size particles is maintained, and the agglomerations are also reduced. This can be explained by the properties of mannitol and similar cryoprotectants, which are able to form a protective matrix around the nanoparticles, isolating them as an unfrozen segment which in turn prevents the particles from agglomeration [31,32].

Eventually, the stabilizers employed in this study are well established in the literature along with other sugars [20]. Hence future studies may investigate the use of such agents in more detail and also consider alternatives. Trehalose, for instance, could possibly decrease the amount of cyroprotectant used, yet trehalose may complicate the composition and activity of the samples and is more expensive than common sugars, an economic aspect which may need to be considered as part of any practical application [20]. Notably, certain sugars may also serve as nutrients, and this may be counterproductive in the context of antimicrobial activity. It is, therefore, worthwhile to investigate alternative stabilizers which may be either more effective, less problematic and perhaps also more readily available. It may even be possible to identify “two-in-one” agents able to substitute simultaneously for both stabilizers, Plantacare^®^ and mannitol, or to venture into agents which are “waste” or themselves biologically active for the additional “kick” [33]. In any case, the NaLyRe sequence represents a major improvement in the production and handling of otherwise insoluble or sparingly soluble materials and, indeed, one may now consider possible medical and agricultural applications in earnest.

From a biological perspective, the initial attempts with mannitol have already enabled lyophilization and resuspension with considerable biological activities observed for the resuspended samples against the microbes tested. Among the chalcogens, tellurium appears to be most toxic overall. It was least effective against *S. cerevisiae*, reducing the growth by just 20% at a formal concentration of 2000 µM (Figure 6). For *C. albicans* this activity was somewhat higher, with significant reductions already at a formal concentration of 1000 µM (Figure 7). The chalcogens were particularly effective against the two bacteria investigated, especially for Gram-positive *S. carnosus* where the growth was reduced by 50% in the presence of 2000 µM for selenium (Figure 8 and Figure 9). In the case of the multicellular nematodes, tellurium was also active and, at a concentration of 200 µM, reduced growth to 60%. While the activity of selenium is often comparable to the one of tellurium, selenium was more active against the nematodes with a reduction of viability to below 50% of the control, while sulfur achieved a reduction to 70% (Figure 10).

In Medicine, such resuspended particles therefore may be employed against topical infections, for instance in the case of skin, mucous, nails and the gastrointestinal tract. Here, the activity of the more active tellurium particles is of special interest. Many organotellurium compounds, as well as simple tellurium salts, often show considerable activity against pathogenic organisms, in some instances even re-sensitizing drug resistant strains against common antibiotics [34]. Yet tellurium is also a fairly toxic element per se, and any application, even a topical one, clearly requires further investigations to exclude any unwanted side effects [35,36]. The particular particulate structure may actually be a benefit rather than drawback in this context, as it almost rules out the kind of systemic uptake and distribution characteristic of soluble, toxic organotellurium compounds and, at the same time, may provide a slowly releasing system for certain reactive tellurium species (RTeS).

General toxicity is less of an issue with the trace element selenium. While the selenium nanoparticles may be oxidized or reduced as well to release diverse reactive selenium species (RSeS), selenides (H_2_Se), selenite (SeO_3_^2−^) and selenate (SeO_4_^2−^) are readily detoxified and even utilized by the human body. The activity of the selenium-based nanosuspensions is therefore rather stimulating, especially in the context of *C. albicans*, as yeasts are commonly known to be rather sensitive against this chalcogen and its diverse compounds. Here, the resuspended selenium particles, at a concentration of 2000 µM, reduce growth by 40%. Indeed, certain anti-dandruff shampoos contain selenium, and chemically speaking, the rather unusual mixed sulfur-selenium ring structures at the centre of such activity are not that different from the Se_8_ rings found in the selenium nanoparticles [37,38,39]. Similarly, the resuspended selenium particles were also active against both strains of bacteria, at a formal concentration of 2000 µM reducing growth of *S. carnosus* to 55%.

In any case, the precise mode(s) of action of these particles, against microbes and also in more complex organisms—from *S. feltiae* to humans—needs to be studied in considerable detail as part of future investigations and also to rule out any undesired or detrimental side effects of this material. Based on the literature available to date and on previous studies, it is feasible that such chalcogen particles act via a combination of mechanisms, which may involve more general physical interactions of the particles with cells and organelles, rather specific surface interactions—such as binding of and to proteins and enzymes, a specific surface chemistry of the chalcogens as well as a slow release of chalcogen-based molecules, such as the kind of inorganic polysulfides (S_x_^2−^) mentioned in the Introduction.

From a wider perspective, and besides possible applications as antimicrobials in Medicine, resuspendable powders of selenium and sulfur may also be of interest in the field of Agriculture. As mentioned briefly in the Introduction, colloidal sulfur has a long tradition in the treatment of grapevines and a similar, perhaps more effective treatment could be envisaged for the nanosuspensions [13,14]. Similarly, selenium may also be applied, obviously under considerably more controlled conditions and in considerably lower amounts. Here, sulfur, as well as selenium, may not only protect the plant from (microbial, nematode) predators, these particles may also enrich the soil, and, by slowly degrading, may serve as a reservoir of an inorganic fertilizer and eventually, in the case of selenium, even as trace element enrichment which may be beneficial along the nutritional chain.

In the medium term, the choice of particles and stabilizers will depend on the specific applications under consideration, and native suspensions—which initially are also sterile due to the manufacturing process—as well as resuspended lyophilized preparations seem to be quite stable and may be considered.

## 5. Conclusions

In summary, and without much suspense, we have been able to demonstrate that resuspension of ready-to-use lyophilized powders of sulfur, selenium and tellurium nanosuspensions on demand results in biologically active suspensions, albeit not solutions. The focus of this investigation has been on the proof-of-principle, and to evaluate the general feasibility of the underlying NaLyRe sequence.

Subsequent studies obviously need to investigate the various aspects of production, stability and wider applications of this approach and its products in considerably more detail. The choice of stabilizers and “cryo-protectants” such as Plantacare^®^ and mannitol, in particular, needs to be considered from the perspective of large-scale production, activity, side-effects, economy and environmental impact. Alternatives, for instance derived from agricultural waste or with specific, desirable biological activities may be of particular interest here [33]. The mode(s) of action also need to be considered in considerably more detail, since these materials may impact on living organisms in various, physical, chemical, biochemical and physiological aspects. Such interactions, or nanotoxicity itself, may result in possible limitations due to adverse side effects. Nonetheless, as the examples of certain anti-dandruff formulations and colloidal sulfur illustrate, there is considerable potential stowed away in the elemental forms of these chalcogens, and unlocking this potential may be possible using nanotechnology.

Admittedly, these elemental forms may appear as rather “primitive” in a more biological context, especially when considering the numerous biologically active sulfur, selenium and tellurium compounds already known. Still, elemental forms have the major advantage that they do not carry the ballast of organic groups which may become modified, released or otherwise problematic. Indeed, the considerable recent interest in biologically active polysulfides, such as the tetrasulfide (S_4_^2−^), demonstrates that “chalcogen only” substances can play a significant role in biological systems [12,40]. One may therefore speculate that S_8_ or Se_8_ rings exposed on the surface of a mighty, at the same time slowly moving and degrading and chalcogen-releasing particle might exhibit considerable activity. Eventually, and with the NaLyRe sequence now available, other materials, such as sparingly or insoluble natural materials or products, parts of plants and even waste, may be processed in the same manner, therefore widening the scope of this approach and providing access to an even wider field of materials and applications [33,41].

## Figures and Tables

**Table 1:** ZP measurements of chalogen nanoparticles. Non-FD or “non-freeze-dried” and FD or “freeze-dried”.

Chalogen	In Original Suspension Medium		In Conductivity-Adjusted Water	
	Non-FD	FD	Non-FD	FD
S	−30 mV	−36 mV	−47 mV	−54 mV
Se	−24 mV	−26 mV	−40 mV	−41 mV
Te	−27 mV	−30 mV	−31 mV	−36 mV
